# Trends in prevalence and mortality burden attributable to smoking, Brazil and federated units, 1990 and 2017

**DOI:** 10.1186/s12963-020-00215-2

**Published:** 2020-09-30

**Authors:** Deborah Carvalho Malta, Luisa Sorio Flor, Ísis Eloah Machado, Mariana Santos Felisbino-Mendes, Luisa Campos Caldeira Brant, Antonio Luiz Pinho Ribeiro, Renato Azeredo Teixeira, Eduardo Marques Macário, Marissa B. Reitsma, Scott Glenn, Mohsen Naghavi, Emmanuela Gakidou

**Affiliations:** 1 Department of Maternal and Child Nursing and Public Health, Nursing School, Universidade Federal de Minas Gerais, Avenida Alfredo Balena, n.° 190, Santa Efigênia, Belo Horizonte, MG CEP: 30130-100 Brazil; 2 Postgraduate Program in Nursing, Nursing School, Universidade Federal de Minas Gerais, Belo Horizonte, MG Brazil; 3 Institute for Health Metrics and Evaluation (IHME), University of Washington, Seattle, WA USA; 4 Department of Family Medicine, Mental and Public Health, School of Medicine, Universidade Federal de Ouro Preto, Ouro Preto, MG Brazil; 5 Department of Internal Medicine, School of Medicine, Universidade Federal de Minas Gerais, Belo Horizonte, MG Brazil; 6 Postgraduate Program in Public Health, School of Medicine, Universidade Federal de Minas Gerais, Belo Horizonte, MG Brazil; 7 Department of Health Analysis and Surveillance of Noncommunicable Diseases, Secretariat of Health Surveillance, Ministry of Health, Brasília, DF Brazil

**Keywords:** Global burden of disease, Quality-adjusted life years, Risk factors, Smoking, Tobacco use

## Abstract

**Background:**

The present study sought to analyze smoking prevalence and smoking-attributable mortality estimates produced by the 2017 Global Burden of Disease Study for Brazil, 26 states, and the Federal District.

**Methods:**

Prevalence of current smokers from 1990 to 2017 by sex and age was estimated using spatiotemporal Gaussian process regression. Population-attributable fractions were calculated for different risk-outcome pairs to generate estimates of smoking-attributable mortality. A cohort analysis of smoking prevalence by birth-year cohort was performed to better understand temporal age patterns in smoking. Smoking-attributable mortality rates were described and analyzed by development at state levels, using the Socio-Demographic Index (SDI). Finally, a decomposition analysis was conducted to evaluate the contribution of different factors to the changes in the number of deaths attributable to smoking between 1990 and 2017.

**Results:**

Between 1990 and 2017, prevalence of smoking in the population (≥ 20 years old) decreased from 35.3 to 11.3% in Brazil. This downward trend was seen for both sexes and in all states, with a marked reduction in exposure to this risk factor in younger cohorts. Smoking-attributable mortality rates decreased by 57.8% (95% UI − 61.2, − 54.1) between 1990 and 2017. Overall, larger reductions were observed in states with higher SDI (Pearson correlation 0.637; *p* < 0.01). In Brazil, smoking remains responsible for a considerable amount of deaths, especially due to cardiovascular diseases and neoplasms.

**Conclusions:**

Brazil has adopted a set of regulatory measures and implemented anti-tobacco policies that, along with improvements in socioeconomic conditions, have contributed to the results presented in the present study. Other regulatory measures need to be implemented to boost a reduction in smoking in order to reach the goals established in the scope of the 2030 United Nations Agenda for Sustainable Development.

## Background

Health risks regarding tobacco consumption are widely documented in the literature [1–3] and are key risk factors for chronic non-communicable diseases (NCD) [1–3], such as cardiovascular conditions [3, 4], cancer (lungs, oral cavity, breast, among others), chronic respiratory diseases, intrauterine growth restriction, and predisposition to premature births. The negative health impact of tobacco results from both the direct consumption of diverse forms of tobacco products (smoked, inhaled, or chewed) and exposure to secondhand smoke [1–5].

Estimates from the Global Burden of Disease (GBD) Study indicate that the global prevalence of current smokers among individuals of 15 years of age and older declined from 27.8% (95% CI, 27.5–28.1%) to 20.1% (95% CI, 19.8–20.4%) between 1990 and 2017. However, population growth and aging have contributed to an increase in disease burden attributable to smoking among middle- and low-income countries. Moreover, smoking continued to be the second leading risk factor for premature death and disability worldwide in 2017 [6].

Several efforts and policies have been coordinated by the World Health Organization (WHO) with the aim of reducing the avoidable adverse effects of tobacco, such as the “Framework Convention on Tobacco Control (FCTC),” in 2003, and the “Global Action Plan for the Prevention and Control of NCDs,” in 2013, both of which targeted the reduction in tobacco use by 30% between 2015 and 2025 [7]. More recently, in 2015, a specific target on tobacco control was included in the 2030 United Nations Agenda for Sustainable Development, seeking to boost public health worldwide [8].

In Brazil, national surveys indicate that the prevalence of current smokers among adults decreased dramatically over the last two decades, from 34.8% in 1989 [9, 10] to 15% in 2013 [11, 12]. This reduction may be attributable to important regulatory measures implemented by the country, especially after the ratification of the FCTC in 2005, such as a national ban on tobacco advertising, a national comprehensive smoke-free policy, large pictorial health warnings on cigarette packages, and continuous raises in taxes and prices of tobacco products [10]. To continue to lower smoking rates, a plan to monitor tobacco use, a list of actions for tobacco control, and national tobacco use reduction goals were added to the country’s “Strategic Action Plan for Coping with NCDs, 2011-2022” [10, 13, 14].

The GBD study, carried out by the Institute for Health Metrics and Evaluation (IHME), innovates by enabling the concomitant evaluation of prevalence and the burden of disease attributed to smoking, both in terms of mortality and non-fatal health outcomes, through the comparative risk assessment (CRA) framework developed by Murray and Lopez [15]. Thus, the present study analyzes trends in current smoker prevalence and mortality attributed to smoking between 1990 and 2017, in Brazil, its 26 states, and the Federal District.

## Methods

This analysis uses data from the 2017 GBD study concerning smoking prevalence and mortality attributable to smoking for Brazil and its states.

### Prevalence estimates

Prevalence of current smokers, defined as individuals who currently use any smoked tobacco product on a daily or occasional basis, was estimated using data from cross-sectional nationally representative surveys. Similarly, the prevalence of former smokers, defined as individuals who quit using all smoked tobacco products for at least 6 months, was also computed and incorporated into the attributable mortality calculation. For Brazil, data from the following surveys were used: (a) the National Survey of Nutrition and Health (PNSN in Portuguese), in 1989, a household survey with a sample of 62,000 respondents [16]; (b) the World Health Survey (WHS), in 2003, a sample in 5000 Brazilians over the age of 18, selected, probabilistically, in 188 municipalities [17]; (c) the Global Adult Tobacco Survey (GATS), included in the National Household Sample Survey (PNAD in Portuguese), conducted by the Brazilian Institute of Geography and Statistics (IBGE in Portuguese) in 2008, whose sample size was 39,425 respondents of 15 years of age or older, and in the present study, only those over 18 years of age were analyzed [12, 18]; (d) the National Health Survey (PNS in Portuguese), a household survey conducted by IBGE, whose sample size was 64,000 respondents over 18 years of age [11, 12], in 2013; (e) the Telephone Survey Surveillance System for Risk and Protective Factors for Chronic Diseases (VIGITEL in Portuguese), an annual survey conducted by the Ministry of Health between 2006 and 2017, carrying out approximately 54,000 interviews in Brazilian capitals among adults of 18 years of age or older [19]. After extracting the data, adjustments for alternative case definitions, as well as for data reported in non-standard age or sex groups, were performed when necessary, enabling a direct comparison between different studies. Finally, current and former smoker prevalence were modeled using the Spatiotemporal Gaussian process regression (ST-GPR), which resulted in a complete time series (1990–2017) for each risk factor for all demographic groups and locations, as described elsewhere [6].

### Attributable fractions

In order to estimate the attributable fractions, initially the risk-outcome pairs are selected by a process of searching the literature, aiming to identify for which outcomes there is an evidence that support the causal relationship between the risk factor and the outcome, as well as quantify the magnitude of associations and uncertainties. Relative risk (RR) estimates derived from prospective cohort studies comparing smokers to never smokers, by cigarettes per smoker per day, pack-years, and years since quitting. These estimates were extracted for all risk-outcome pairs, identified as being caused by smoking (tuberculosis, infections of the lower respiratory tract, esophageal cancer, stomach cancer, bladder cancer, liver cancer, larynx cancer, lung cancer, breast cancer, cervical cancer, colorectal cancer, lip and mouth cancer, nasopharyngeal cancer, pharynx cancer, pancreatic cancer, kidney cancer, leukemia, ischemic heart disease, ischemic stroke, hemorrhagic stroke, subarachnoid hemorrhage, fibrillation and flutter, aortic aneurysm, peripheral arterial disease, chronic obstructive pulmonary disease, other chronic respiratory diseases, asthma, peptic ulcer, gallbladder and biliary tract, Alzheimer’s disease and other dementias, Parkinson’s disease (protection), multiple sclerosis, type II diabetes, rheumatoid arthritis, lower back pain, cataract, macular degeneration, and fracture). For each of the outcomes, non-linear dose-response curves were produced, by sex and age, using a Bayesian meta-regression model. Risk curves of former smokers in relation to those who had never smoked were also estimated [6].

Population-attributable fractions were calculated based on estimates of exposure, relative risks, and the theoretical minimum risk exposure level (TMREL) for smoking (zero smoking). The TMREL is based on the assumption that, if in the past the population exposure had been modified for a level of theoretical minimum risk of exposure, this would result in a minor loss of health [20, 21].

### Data analysis

#### Prevalence

Prevalence of current smokers is presented for Brazil and its states, by sex, from 1990 to 2017. Additionally, GBD estimates were compared to adult (≥ 18 years old) smoking prevalence obtained from different national household surveys [11, 16–18] and telephone-based surveys [19]. Finally, a cohort analysis of smoking prevalence by birth-year cohort was performed to better understand temporal age patterns in smoking.

#### Attributable mortality

Estimates of deaths were multiplied by outcome-specific population-attributable fractions (PAF) and then summed across all outcomes to compute overall mortality attributable to smoking. The absolute number of deaths attributable to smoking and the relative percentage of change between the two periods (1990–2017) are reported here for all causes and for cardiovascular diseases, neoplasms, diabetes, chronic respiratory diseases, digestive diseases, neurological disorders, and muscle skeletal disorders. Cardiovascular diseases and neoplasms were later disaggregated for an expanded analysis.

Relative changes in mortality rates were also described by the Socio-Demographic Index (SDI). The SDI is a summarized measure of the socio-demographic development of a specific location and is based on the average lag-distributed income per capita, on the average educational attainment among individuals of 15 years of age or older and on the total fertility rate (TFR). This index has an interpretable scale which varies from zero to one, where zero represents the lower per capita income, lower level of education, and higher observed TFR and one represents the higher per capita income, higher level of education, and lower TFR [20].

Finally, to understand the drivers of changes in mortality attributable to smoking, from 1990 to 2017, the present study investigated the relative contribution of the following factors: (1) population growth, (2) population age structure, (3) exposure to smoking, and (4) risk-deleted rates. Risk-deleted rates are defined as the mortality rates that would have been recorded if smoking had been removed as a risk factor. Methods used for the decomposition analysis were developed by Das Gupta (1993) [22] and are detailed in a previous publication [15].

### Uncertainty analysis

The analytical process of generating point values for GBD indicators also requires an estimate of the uncertainty interval (UI) as a function of the variability caused by sampling errors, uncertainties in the coefficients of statistical modeling, and model life table systems, among other methodological processes. Thus, GBD provides the uncertainty range for its key estimates. Simulations of 1000-metric samples are produced by location, sex, age, and all years covered by each analytical step of the posterior distribution in the estimation process. Further details can be read in other studies [23].

### Ethical considerations

The GBD Brazil Project was approved by the Ethics Committee on Research from the Federal University of Minas Gerais (UFMG in Portuguese), logged under Protocol Number 62803316.7.0000.5149.

## Results

### Current smoking prevalence

**Fig. 1:**
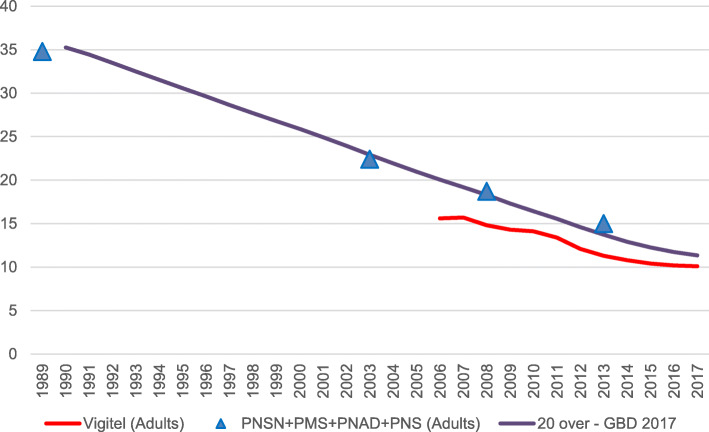
Current smoking prevalence trends for Brazil according to GBD 2017 estimates (purple line) for 20 plus population from 1990 to 2017, Brazilian National Household Surveys’ crude values (blue triangles) for the 18 plus population in 1989, 2004, 2008, and 2013, and Vigitel crude values (red line) for the 18 plus population from 2006 to 2017. Estimations are no age-standardized. Sources: Brazilian Surveys in adults (PNSN 1989 [9], PMS–2004 [9], PNAD (GATS)–2009 [12, 18], and PNS–2013 [11, 12]; Vigitel (2006 to 2017) [10, 19]

Figure 1 shows GBD smoking prevalence estimates for adults of 20 years of age or older, together with the prevalence obtained from the main national household surveys and the Vigitel telephone-based survey for those participants of 18 years of age or older. According to the GBD, a significant decrease in smoking prevalence in the adult population (> 20 years old) was achieved in Brazil between 1990 and 2017, from 35.3% (95% UI 32.9, 37.8) to 11.3% (95% UI 10.4, 12.4), respectively. This trend is similar to that found in other national studies.

Table 1 shows the age-standardized (≥ 20 years old) prevalence of current smokers and the annualized change in prevalence between 1990 and 2017, by sex, for Brazil and each of the states. Smoking rate among men is approximately 1.5 times higher than that among women in the country. In 2017, the state of São Paulo presented the highest prevalence for males (17.9% [95% UI 16.1, 19.7]), followed by Rio Grande do Sul (17.1% [UI 95% 15.0, 19.2]). The highest rates among females were also found in these states, at 12.6% (UI 95% 10.1, 15.4) in Rio Grande do Sul and 11.7% (UI 95% 9.6, 14.2) in São Paulo. The annualized percent change between 1990 and 2017 was − 4.5% (95% UI − 5.2, − 3.8) among women and − 3.8% (95% UI − 4.3, − 3.4) among men (Table 1).

**Table 1:** Age-standardized current smoker prevalence estimates, according to sex, and annualized percent change between 1990 and 2017 for Brazil and its states

Local	Age-standardized prevalence 2017		Annualized percent change	
			1990–2017	
	Female	Male	Female	Male
Brazil	8.7 (7.4, 10.3)	13.9 (12.9, 15.1)	− 4.5 (− 5.2, − 3.8)	− 3.8 (− 4.3, − 3.4)
Acre	9.1 (7.1, 11.4)	12.5 (11, 14.4)	− 4.7 (− 5.9, − 3.4)	− 4.2 (− 4.8, − 3.5)
Alagoas	5.4 (4.1, 7)	11 (9.6, 12.4)	− 5.1 (− 6.5, − 3.7)	− 4.1 (− 4.9, − 3.4)
Amazonas	5.3 (4.1, 7)	11 (9.5, 12.5)	− 4.6 (− 6.1, − 3)	− 4.1 (− 4.9, − 3.4)
Amapá	5.8 (4.4, 7.5)	11.9 (10.3, 13.6)	− 4.7 (− 6.1, − 3.2)	− 4.1 (− 4.8, − 3.4)
Bahia	5 (3.8, 6.4)	8.7 (7.6, 10)	− 5.3 (− 6.8, − 3.9)	− 4.6 (− 5.3, − 3.9)
Ceará	5.5 (4.2, 7.2)	10.8 (9.4, 12.3)	− 5.3 (− 6.6, − 3.9)	− 4.4 (− 5, − 3.6)
Distrito Federal	8.1 (6.3, 10.3)	12.5 (10.9, 14.2)	− 4.4 (− 5.7, − 3.1)	− 3.8 (− 4.6, − 3.1)
Espírito Santo	6.7 (5.2, 8.7)	12.1 (10.7, 13.6)	− 5.2 (− 6.5, − 4)	− 4.1 (− 4.8, − 3.4)
Goiás	6.9 (5.4, 8.8)	13.4 (11.7, 15.2)	− 5 (− 6.1, − 3.6)	− 3.8 (− 4.5, − 3.1)
Maranhão	4.1 (3, 5.4)	9.9 (8.6, 11.3)	− 5.3 (− 6.8, − 3.7)	− 4.4 (− 5.2, − 3.6)
Minas Gerais	9.2 (7.3, 11.5)	15 (13.3, 16.9)	− 4.2 (− 5.4, − 3)	− 3.6 (− 4.3, − 3)
Mato Grosso do Sul	8 (6.2, 10.2)	14.4 (12.6, 16.3)	− 4.4 (− 5.7, − 3)	− 3.8 (− 4.5, − 3.1)
Mato Grosso	6.7 (5.2, 8.5)	13.3 (11.7, 15.2)	− 4.8 (− 6.1, − 3.4)	− 3.8 (− 4.5, − 3.2)
Pará	6 (4.5, 7.8)	11.5 (10, 13.1)	− 4.7 (− 6.1, − 3.2)	− 4.2 (− 5, − 3.5)
Paraíba	5.7 (4.3, 7.3)	11.9 (10.5, 13.6)	− 4.6 (− 5.9, − 3.2)	− 4.1 (− 4.8, − 3.3)
Paraná	11 (8.7, 13.7)	16.1 (14.4, 18.2)	− 4.4 (− 5.5, − 3.2)	− 3.6 (− 4.2, − 3)
Pernambuco	8.5 (6.5, 10.7)	12.6 (11, 14.4)	− 3.9 (− 5.2, − 2.5)	− 3.9 (− 4.5, − 3.1)
Piaui	5.7 (4.3, 7.5)	11 (9.6, 12.7)	− 4.9 (− 6.4, − 3.4)	− 4.4 (− 5.1, − 3.7)
Rio de Janeiro	9.4 (7.4, 11.6)	12.9 (11.3, 14.7)	− 4.3 (− 5.4, − 3.1)	− 3.6 (− 4.3, − 3)
Rio Grande do Norte	6.1 (4.7, 7.7)	11 (9.5, 12.5)	− 4.9 (− 6.2, − 3.4)	− 4.2 (− 4.9, − 3.5)
Rondônia	7.6 (5.9, 9.6)	12.2 (10.7, 13.9)	− 4.7 (− 6.1, − 3.3)	− 4.1 (− 4.8, − 3.4)
Roraima	5.5 (4.2, 7)	11.2 (9.8, 12.8)	− 5.2 (− 6.5, − 3.8)	− 4.6 (− 5.3, − 4)
Rio Grande do Sul	12.6 (10.1, 15.4)	17.1 (15, 19.2)	− 4.5 (− 5.5, − 3.6)	− 3.7 (− 4.3, − 3)
Santa Catarina	10.2 (8, 13)	13.7 (11.9, 15.7)	− 4.5 (− 5.7, − 3.3)	− 4.1 (− 4.7, − 3.4)
Sergipe	4.3 (3.2, 5.7)	10.4 (9, 12.1)	− 4.9 (− 6.5, − 3.2)	− 4 (− 4.8, − 3.2)
São Paulo	11.7 (9.6, 14.2)	17.9 (16.1, 19.7)	− 4 (− 4.9, − 3)	− 3.4 (− 4, − 2.9)
Tocantins	6 (4.4, 7.9)	10.9 (9.5, 12.4)	− 4.6 (− 6.1, − 3)	− 4.1 (− 4.8, − 3.4)

**Fig. 2:**
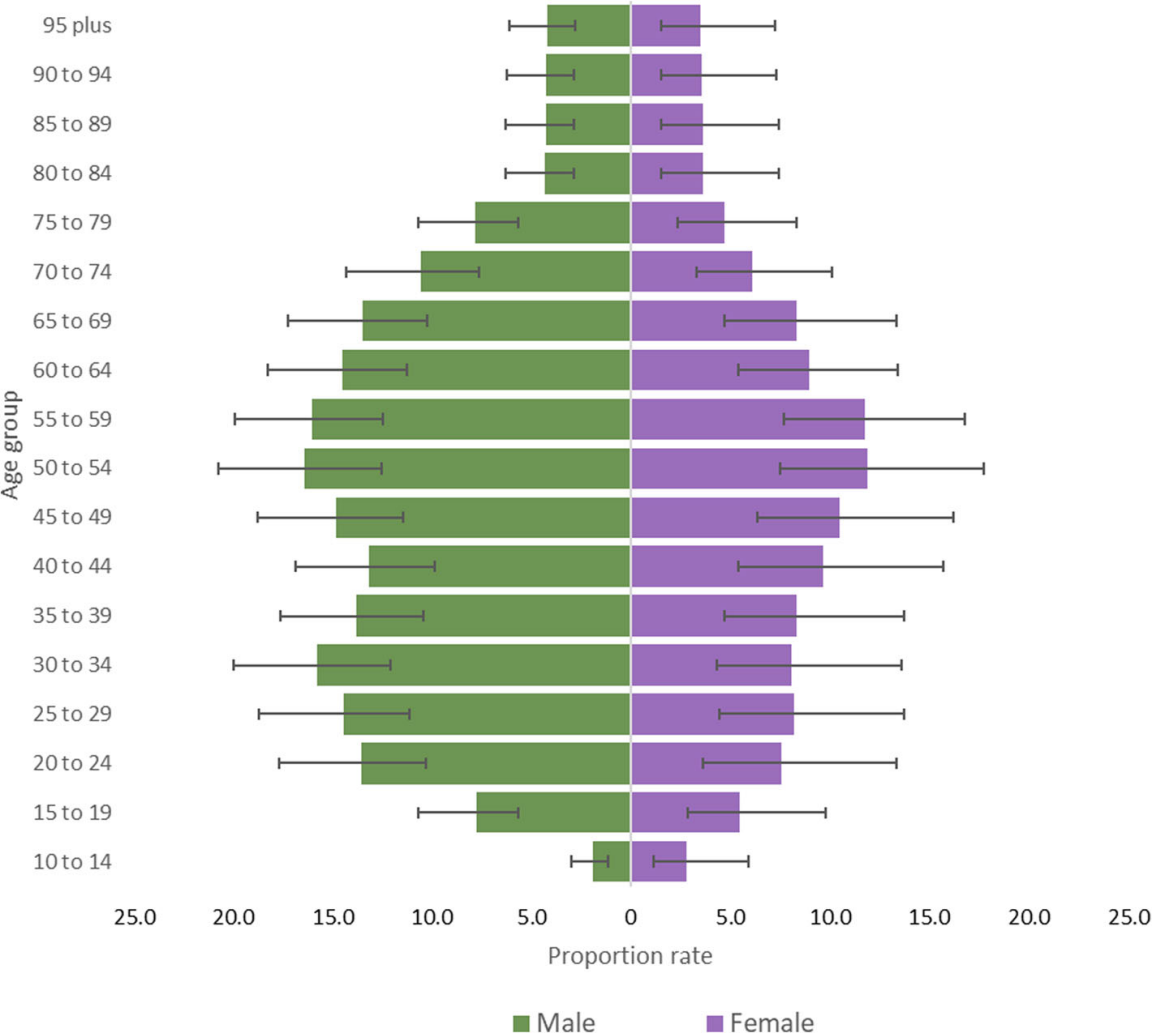
The colored bars represent the prevalence of current smokers by each age group defined at the y axes in Brazil in 2017. Men are on the left (green) and women on the right (purple)

In Brazil, smoking prevalence peaks between the ages of 50 and 54 years for both sexes, exceeding 15% among men and 10% among women in this age group. Reduced rates are observed for those in the most extreme age groups. Smoking prevalence among young male adults aged 20–24 is about twice as high as the rates seen among those aged 15–19, at 13.6% (95% UI 10.3, 17.7) and 7.8% (95% UI 5.6, 10.6), respectively (Fig. 2).

**Fig. 3:**
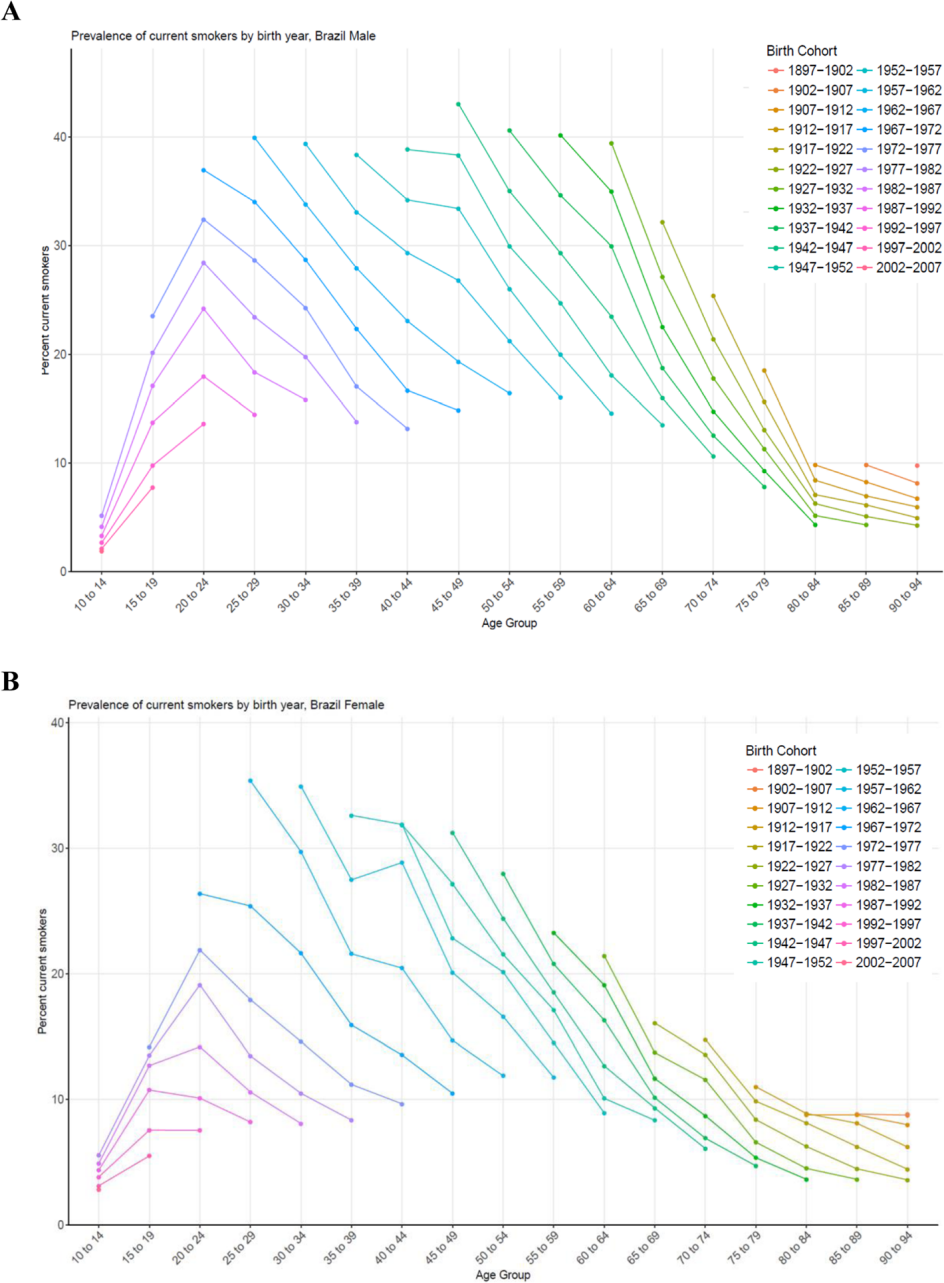
Prevalence of current smokers by age group, and birth-year cohort in Brazil for men (**a**) and women (**b**)

Across birth cohorts, smoking prevalence decreased by age group and sex (Fig. 3). Sizeable reductions in smoking prevalence in 20 to 24 years old occurred across birth cohorts. For women, prevalence is consistently lower than for men; nevertheless, reductions in smoking prevalence across birth cohorts were generally smaller than those recorded for men.

### Mortality attributable to smoking

**Fig. 4:**
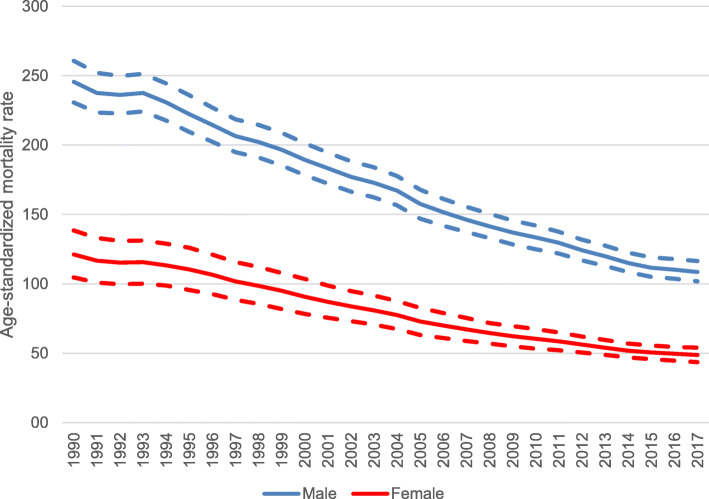
Age- standardized mortality rate (per 100,000 inhabitants) by all causes attributable to smoking for men (blue line) and women (red line) and uncertainty intervals (dashed lines) in Brazil, from 1990 to 2017

Between 1990 and 2017, mortality rates attributable to smoking decreased for both sexes in Brazil (Fig. 4). A larger decrease was observed for females (− 59.8% [95% UI − 65.0, − 52.8]) compared to males (− 55.8% [95% UI − 59.0, − 52.4]).

Table 2 shows the absolute number of deaths and the age-standardized mortality rate (per 100,000 inhabitants) attributable to smoking, as well as the relative variation in mortality rates between 1990 and 2017, for Brazil and each state. At the national level, the all-cause age-standardized mortality rate declined from 177.5/100,000 inhabitants (95% UI 164.6, 191.5) to 74.9/100,000 inhabitants (95% UI 69.7, 80.8) in the analyzed period, a relative reduction of 57.8% (95% UI 61.2, 54.1). Rates have declined in all states, and in 2017, the highest mortality rate attributable to smoking was found in Pernambuco (93.7/100,000 [95% UI 82.0, 107.8]), followed by Acre (89.5/100,000 [95% UI 76.9, 102.9]) and Rio Grande do Sul (83.7/100,000 [95% UI 71.6, 96.1]). On the other hand, rates lower than the national level were observed in several states, such as in the Federal District (59.6/100,000 [95% UI 49.0, 71.6]), Piauí (61.8/100,000 [95% UI 53.2, 71.1]), and Tocantins (62.3/100,000 [95% UI 52.4, 73.2]).

**Table 2:** Deaths, age-standardized mortality rates per 100,000 attributed to smoking, and mortality rate percent change in the period of 1990 to 2017, Brazil and its states

Locality	1990				2017				% Change rate	95% UI
	Deaths	95% UI	Rate	95% UI	Deaths	95% UI	Rate	95% UI		
Brazil	152,554	(142,793, 163,137)	177.5	(164.6, 191.5)	167,657	(156,056, 180,408)	74.9	(69.7, 80.8)	− 57.8	(− 54.1, − 61.2)
Acre	277	(244, 307)	179.1	(156.5, 201)	492	(423, 565)	89.5	(76.9, 102.9)	− 50.0	(− 42.1, − 56.7)
Alagoas	2160	(1893, 2429)	162.4	(141.5, 182.9)	2387	(2023, 2734)	79.0	(67, 90.6)	− 51.4	(− 43.5, − 58.8)
Amapá	120	(104, 136)	130.0	(110.5, 151.1)	315	(265, 363)	69.2	(58.6, 79.8)	− 46.8	(− 36.4, − 54.9)
Amazonas	1066	(940, 1201)	142.8	(125.1, 162.1)	1905	(1653, 2153)	75.8	(65.8, 85.8)	− 46.9	(− 38.5, − 54.3)
Bahia	8882	(7680, 10,062)	131.2	(113.5, 149.1)	11,332	(9897, 12,742)	72.1	(63, 81)	− 45.0	(− 36.3, − 52.9)
Ceará	5095	(4511, 5727)	126.0	(111.3, 141.9)	6908	(5986, 7866)	70.8	(61.5, 80.5)	− 43.8	(− 34.6, − 51.8)
Distrito Federal	771	(646, 899)	135.8	(111.6, 160)	1259	(1051, 1480)	59.6	(49, 71.6)	− 56.1	(− 44.9, − 63.9)
Espírito Santo	2402	(2116, 2682)	181.0	(158.9, 205.3)	2669	(2309, 3065)	63.8	(55.1, 73.5)	− 64.8	(− 58.6, − 69.8)
Goiás	3030	(2673, 3389)	167.3	(145.7, 190.1)	4619	(3937, 5304)	72.3	(61.7, 82.9)	− 56.8	(− 49, − 62.9)
Maranhão	3589	(3199, 4008)	137.5	(122.3, 155)	4286	(3714, 4874)	68.2	(59.1, 77.6)	− 50.4	(− 42.3, − 57.3)
Mato Grosso	1136	(975, 1281)	150.6	(128.9, 171.9)	2085	(1779, 2377)	71.4	(60.9, 81.3)	− 52.6	(− 43.8, − 59.6)
Mato Grosso do Sul	1451	(1268, 1639)	174.1	(149.9, 198.6)	2089	(1773, 2437)	77.0	(65.2, 90)	− 55.8	(− 48.4, − 62.5)
Minas Gerais	16,996	(14,990, 18,837)	178.8	(156.1, 200.7)	17,265	(14,958, 19,684)	68.0	(58.9, 77.7)	− 62.0	(− 55.7, − 67.5)
Pará	2943	(2553, 3329)	143.0	(123.7, 162.5)	4783	(4151, 5448)	75.5	(65.3, 85.8)	− 47.2	(− 38.9, − 54.9)
Paraíba	3365	(2964, 3784)	148.0	(130.3, 166.5)	3611	(3104, 4160)	78.3	(67.4, 90.1)	− 47.1	(− 37.6, − 55)
Paraná	9401	(8171, 10,614)	209.6	(180.9, 238.8)	9970	(8640, 11,424)	79.9	(69.2, 91.8)	− 61.9	(− 55.9, − 66.9)
Pernambuco	7893	(6936, 8847)	183.0	(159, 207.8)	9105	(7952, 10,445)	93.7	(82, 107.8)	− 48.8	(− 40.1, − 55.7)
Piauí	1880	(1638, 2102)	134.4	(117.2, 150.8)	2214	(1901, 2544)	61.8	(53.2, 71.1)	− 54.0	(− 45.8, − 61)
Rio de Janeiro	20,499	(18,038, 22,999)	218.0	(189.3, 247.4)	17,829	(15,276, 20,622)	81.9	(70, 94.7)	− 62.4	(− 55.5, − 68.5)
Rio Grande do Norte	1956	(1716, 2197)	121.8	(106.6, 136.9)	2623	(2278, 3008)	70.9	(61.8, 81.3)	− 41.8	(− 31.7, − 50.2)
Rio Grande do Sul	13,253	(11,545, 14,815)	212.7	(182.6, 241.7)	12,656	(10,856, 14,504)	83.7	(71.6, 96.1)	− 60.6	(− 53.9, − 66.1)
Rondônia	614	(528, 703)	176.0	(148.8, 204.1)	1062	(880, 1258)	77.0	(63.9, 91.7)	− 56.3	(− 46.7, − 63.9)
Roraima	91	(79, 105)	177.2	(151.6, 206.2)	223	(185, 267)	73.5	(60.5, 88)	− 58.5	(− 49.2, − 66.1)
São Paulo	37,593	(33,090, 41,939)	197.4	(171.8, 223.2)	38,345	(32,955, 43,723)	74.3	(63.7, 85.1)	− 62.4	(− 56.1, − 67.8)
Santa Catarina	4500	(3906, 5031)	190.3	(164.6, 216.5)	5424	(4691, 6177)	71.7	(62, 81.9)	− 62.3	(− 56.5, − 67.9)
Sergipe	1074	(937, 1208)	128.2	(111.8, 144.5)	1359	(1169, 1540)	64.8	(55.6, 73.7)	− 49.5	(− 40.8, − 57.1)
Tocantins	517	(435, 598)	140.6	(118.5, 162.3)	843	(708, 990)	62.3	(52.4, 73.2)	− 55.7	(− 46.2, − 63.1)

**Fig. 5:**
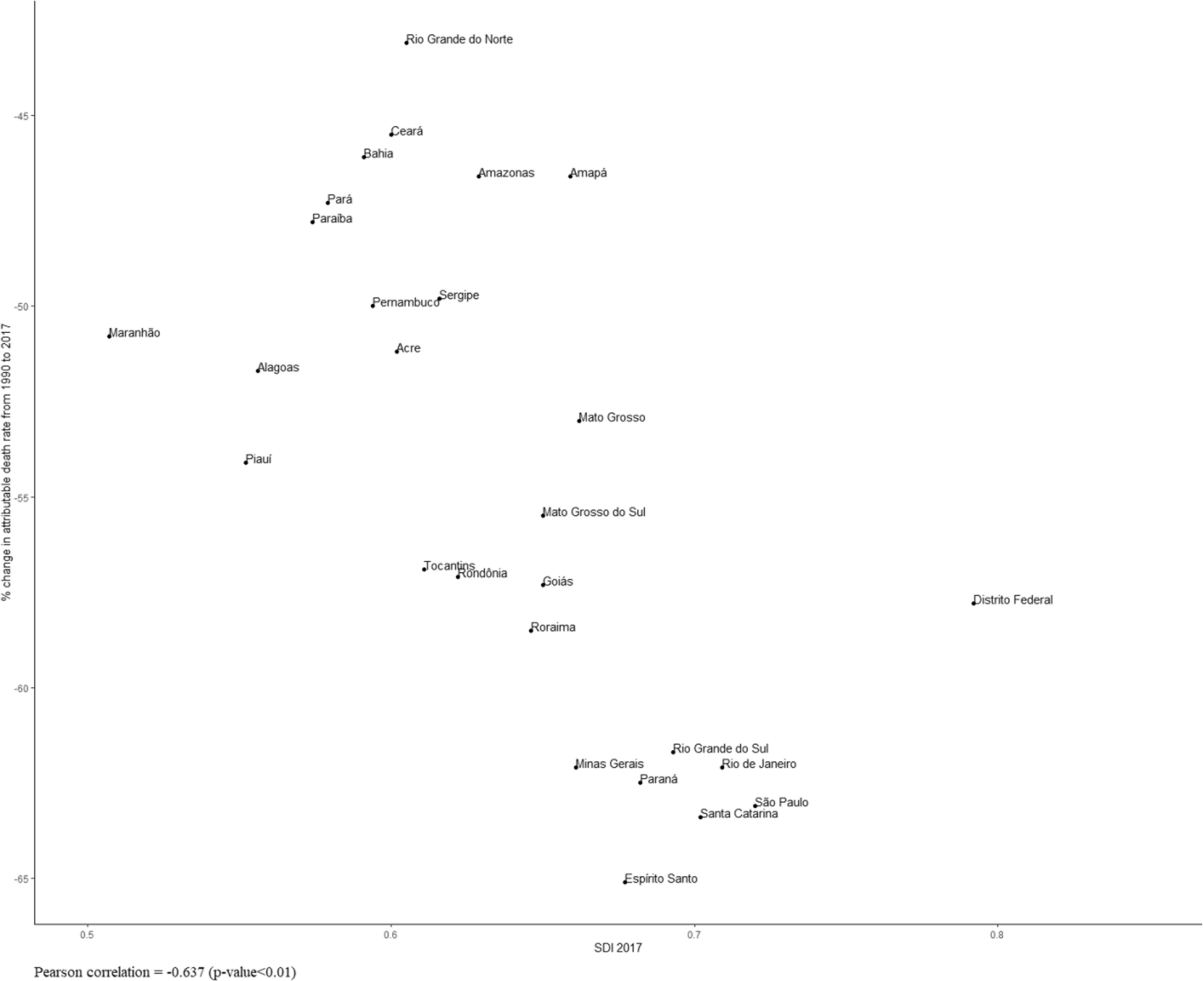
Correlation between the Socio-demographic Index (SDI) (x-axis) and relative change (%) in mortality rates attributable to smoking between 1990 and 2017 in Brazil (y-axis)

Figure 5 shows the relative change in smoking-attributable mortality rates between 1990 and 2017 for each of the states according to SDI. The highest declines were observed for those states with high SDIs, such as Espírito Santo, Santa Catarina, São Paulo, Paraná, Rio de Janeiro, and Minas Gerais. Conversely, smaller variations occurred in states with lower SDIs, such as Rio Grande do Norte, Ceará, Bahia, Pará, and Paraíba, states located in the North and Northeast regions of Brazil (Pearson correlation − 0.637; *p* < 0.001).

**Fig. 6:**
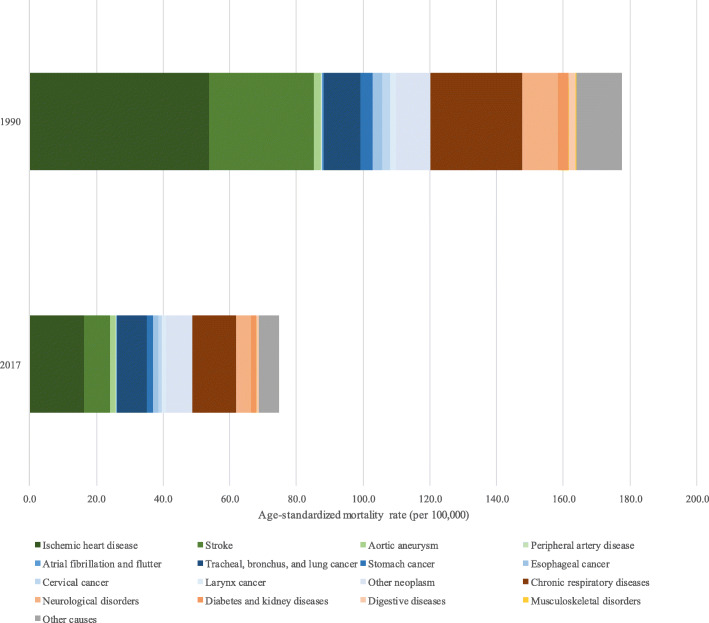
Colored bars are the attributable age-standardized mortality rates attributable to smoking for both sexes, each color a specific cause, across all Brazilian states, 1990 and 2017

The analysis of specific causes of deaths attributable to smoking indicates that mortality due to cardiovascular diseases decreased from 88.0/100,000 inhabitants (95% UI 81.3, 94.3) to 26.3/100,000 inhabitants (95% UI 23.8, 28.9) between 1990 and 2017. Among this group of conditions, the greatest reduction in mortality was observed for stroke (− 75.3%). Death rates due to neoplasia diminished from 32.2/100,000 (95% UI 29.8, 34.6) to 22.6/100,000 (95% UI 21.2, 24.1) in the same period, with the largest relative decreases seen for cervical and stomach cancer, − 60.8% and − 57.3%, respectively. Chronic respiratory disease mortality rates were also lower in 2017 when compared to those in 1990, 27.5/100,000 (95% UI 24.8, 30.2) and 13.0/100,000 (95% UI 11.7, 14.5), respectively (Fig. 6 and Supplementary Table 1). Other causes of death attributed to smoking included diabetes, neurological disorders, diseases of the digestive tract, musculoskeletal disorders, tuberculosis, and other respiratory infections.

**Fig. 7:**
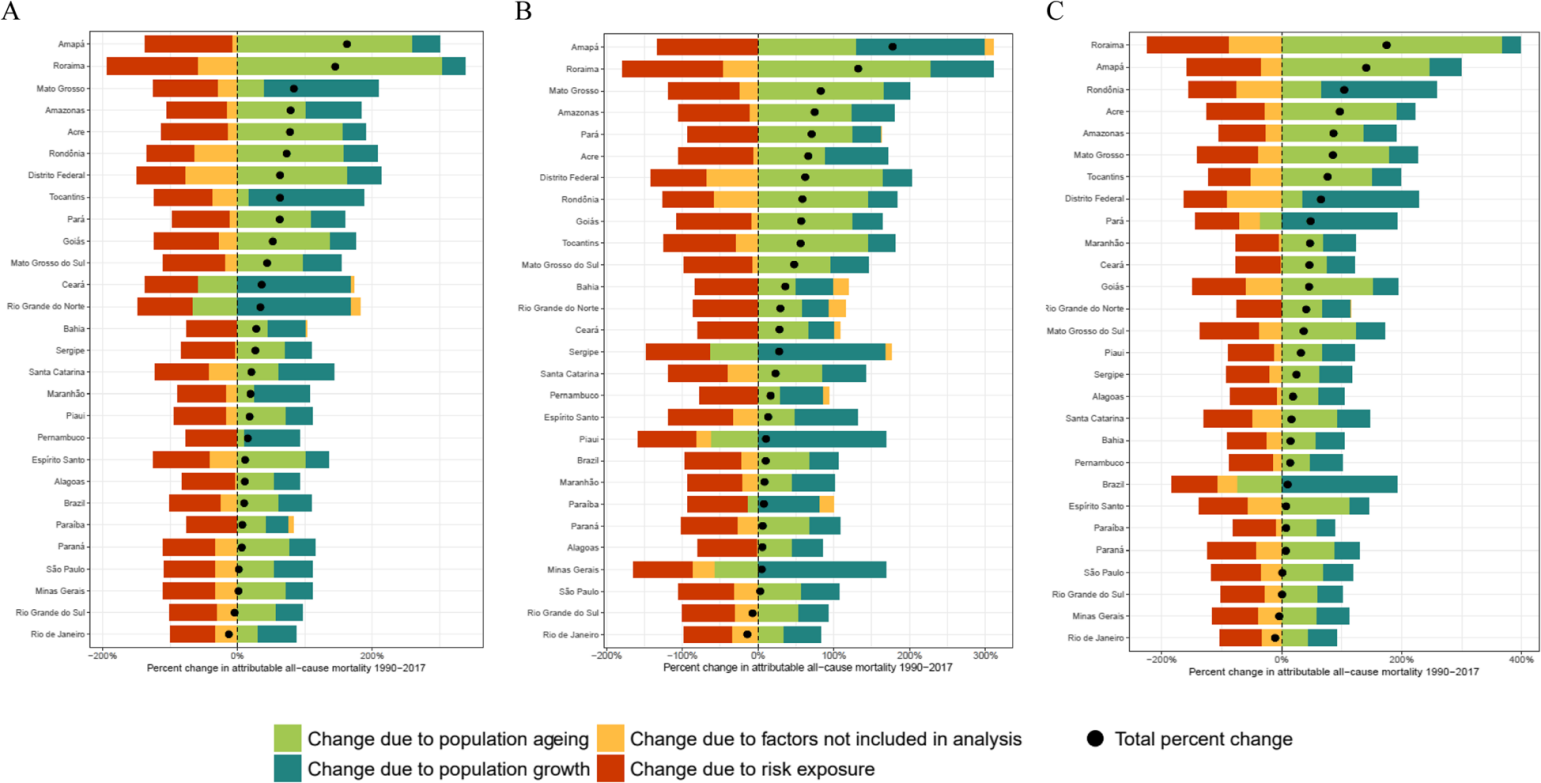
Decomposition of deaths attributable to smoking, both sexes (A), male (B) and female (C). Brazil, 1990 to 2017. Legend: Percent change in smoking-attributable deaths for both sexes (**a**), male (**b**), and female (**c**). Results are shown for all causes combined. The black dot shows total percentage change. The risk-deleted rate is the expected mortality rate if the exposure level for smoking were reduced to the theoretical minimum risk exposure level

Despite the reduction of all-cause smoking-attributable mortality rates in Brazil, the number of deaths rose from 152,554 in 1990 to 167,657 in 2017. This was mainly driven by a combination of population growth and aging and was observed for all states and both sexes, with a more prominent increase in Amapá and Roraima (Fig. 7). Rio de Janeiro and Rio Grande do Sul showed an opposite trend, as a fall in the number of deaths attributable to smoking was observed between 1990 and 2017, mainly due to a reduction in risk exposure. The contributions of each of the factors are fully described in Supplementary Table 2.

## Discussion

The estimates of the 2017 Global Burden of Diseases Study show a decline in the smoking prevalence for both sexes and all age groups from 1990 to 2017, especially in the younger cohorts. In 2017, smoking in Brazil was responsible for almost two hundred thousand deaths. The absolute number of deaths increased, mainly due to population aging and population growth, but the important decrease in mortality rates attributable to smoking is due to the risk-deleted rates. A fall in mortality rates attributable to smoking was also observed, especially related to cardiovascular diseases in the studied period. Expressive progress was made in the states with better socio-demographic conditions, although these states maintained high mortality rates that were attributable to smoking. Our results confirm that the country has made progress in the commitments with the national goals and the global reduction of smoking.

Estimates from the GBD 2017 showed that the reduction in smoking occurred globally and in most countries [6]. In 2000, the WHO reported that 33.3% of the global population of 15 years of age or older were current users of some form of tobacco, while in 2015, this rate had declined to approximately one quarter (24.9%) of the global population [24]. Several studies have shown Brazil among the countries with the greatest relative reduction in prevalence among males and females [3, 6, 16, 25]. These data are consistent with studies conducted by the WHO [24, 26] and the Center for Disease Control (CDC), using the Global Adult Tobacco Survey (GATS) questionnaire, applied in Brazil in 2008, which compared the situation of smoking in 16 countries. In countries participating in GATS, 48.6% (95% CI 47.6–49.6) of the men and 11.3% (10.7–12.0) of the women were tobacco users. The prevalence of current smokers in young patients of 15 years of age or older were as follows: China 52.9%, (50.6–55.2), Russia 60.2 (58.4–62.0), Thailand 45.6 (43.8–47.4, Bangladesh 44.7 (42.5–47.0), Egypt 37.6 (36.3–39.0), India 24.3 (23.3–25·3), Mexico 24.8 (23.2–26.6), the Philippines 47.6 (45.7–49.6), Poland 36.9 (34.9–38.9), Turkey 47.9 (45·9–50·0), Ukraine 50.0 (48.1–52.0), Vietnam 47.4 (45.4–49.4), Uruguay 30.7 (28.2–33.4), the United Kingdom 22.8 (21.6–24.2), and a smaller prevalence was presented in Brazil 21.6 (20.8–22.4) [25]. These studies reinforce the leadership of Brazil as an example in the control of the tobacco use worldwide.

Gender differences are important, and in most countries, men generally smoke more than women. A higher smoking prevalence among women occurred in Europe (20%) [4], followed by the Americas, where men smoke around 1.5 times more than women [4]. Countries, such as Egypt, India, and Bangladesh, with culture and religion marked by great inequality between genders, the smoking prevalence among women proved to be extremely low, lower than 2%, contrasting to the prevalence of approximately 30% among men [25]. The present study’s results found in Brazil repeat the global trend, with a higher prevalence among men, as what has been explained by historic and cultural contexts, by sexism, and by the intense propaganda of the tobacco industries, associating tobacco products with the image of strength, virility, and power [10, 27]. In the western world, and in Brazil, the initiation of smoking among women occurred more recently, around the 1960s and the 1970s, with intense advertising from the tobacco industry, associated with the image of feminine emancipation and gender equality [27–29]. However, in recent decades, this tendency modified itself and the image of tobacco began to be associated with death, generating a subsequent decline in both sexes and age groups [10].

Patterns of smoking differ across cohorts. The more recent cohorts registered a lower prevalence than did those from previous cohorts, which indicates the successful progress of the policies implemented in an attempt to reduce the number of people who begin smoking, such as advertisement bans, higher taxes on tobacco products, health warnings, and a rise in cigarette taxes [10, 12, 14]. These differences between sexes can also be verified by the differences in this study, especially as regards the birth and sex cohorts. The prevalence is higher among men in all cohorts, while a fall in the prevalence in both sexes was observed in the more recent cohorts. This study also found that higher mortality rates among men were decreasing, demonstrating that the smoking habit was higher among men in the past, thus justifying the differences in the magnitude of these rates by sex [30, 31].

Our subnational analysis has shown downward trends in all Brazilian states, highlighting a higher smoking prevalence in the states of the South and Southeast regions, such as Rio Grande do Sul, Parana, Santa Catarina, and São Paulo, as well as cultural aspects, facilitated by the intense presence of immigration in the past, assimilating habits of these populations in previous decades [32, 33]. Brazil is the second largest producer and largest exporter of tobacco in the world, with much of the tobacco industry strongly concentrated in the state of Rio Grande do Sul, while Alagoas is a major producer of twist tobacco, widely used in hand-rolled cigarettes, which may explain the leadership of these states in prevalence [34, 35]. States, such as Acre, also showed a prevalence of high smokers in the PNS, which may be related to border areas with other countries and a subsequent access to cheaper cigarettes through illegal trade and less supervision [12, 18].

However, when analyzing the mortality rates attributable to smoking by SDI, the Southern and Southeastern states are the leaders, showing that despite the downward trend in these rates, a high death rate remains in the locations with better economic development. A possible explanation for this finding may be the low prevalence of smoking among women in the North and Northeast states, which had the lowest SDI and the most agrarian economy in recent decades. As smoking exposure in the country among women was late, the habit became common among women in the 1970s in the more industrialized Southern and Southeastern states at the same time that they were entering the labor market and fighting for gender equality [32, 33]. Thus, in the states of lower SDI, this practice did not reach their women, since, in the following decades, the educational messages of smoking damages became commonplace, and women left the smoking practice.

The 2017 GBD Study shows that smoking occupies the second leading risk factor. Additionally, in Brazil, in the 1990s, smoking was the first risk factor, which is explained by the high prevalence at the time (34.8% in 1986). Brazil achieved a significant reduction in early cigarette cessation, as evidenced by its sharp decline in prevalence in the 20–24 age group [10, 14]. This important reduction in the prevalence and the mortality attributable to tobacco has been explained by the regulatory measures implemented in recent decades [32, 34].

This study also observed an intense decline in mortality caused by cardiovascular disease, cancer, and chronic respiratory illness due to the reduced exposure to the risk of tobacco in the country, reflecting the implementation of regulatory measures. The beneficial effects of smoking cessation on the pathophysiology of cardiovascular diseases occurs rapidly after smoking cessation, with a significant reduction in causes, such as ischemia and other diseases, which explains the decline in nearly 75% of the burden of cardiovascular diseases [3]. On the other hand, smoking’s effects on cancer are observed more slowly. Studies on the association between smoking and lung cancer were first suggested in England in 1927 [35]. Further studies have shown that smoking cessation reduces the risk of lung cancer by pointing out that lung cancer incidence rates in a given country reflect the prevalence of cigarette smoking in the population [29, 35]. Other studies indicate that the maintenance of high mortality rates in older populations is due to the experience of smoking in the past [15, 33, 36].

The monitoring of tobacco indicators in countries is a real necessity, especially as regards the reduction targets established in the WHO National Plan [37], in Global NCDs [7], as well as in United Nations (UN) Sustainable Development Goals (SDG) [8]. However, over one hundred countries worldwide still lack monitoring initiatives, making it difficult to accurately compare and monitor trends in tobacco consumption [38]. It is worth noting that the country organized its Surveillance System for Risk Factors and Protection for Chronic Diseases, conducting household surveys every five years, such as the Global Adult Tobacco Survey in 2008 [18], the National Health Survey in 2013 [11], the annual telephone surveys since 2006 in major capitals (VIGITEL) [19], among others [38]. The best evidence comes from household surveys in adults, as they represent the whole adult population in the country and surveys conducted with schoolchildren. These surveys allow for the constant monitoring of smoking prevalence and the evaluation of the effect of the measures adopted to reduce it [9, 10].

Brazil has been internationally recognized for actions in the field of regulation, education, prevention, and governance toward smoking control [25, 39]. The regulatory measures adopted are in line with cost-effective interventions in the prevention of NCDs published by the WHO [40], such as (a) the increase in taxes and prices on tobacco products, (b) prohibiting smoking in public places, (c) the inclusion of warnings regarding the dangers of tobacco use, and (d) prohibition of tobacco advertising, sponsorship, and promotion. The ban on partial advertising of tobacco products dates back to 1996, followed by a number of measures, such as the ratification of the 2005 Framework Convention on Tobacco Control [5, 10]. Among the recent regulatory measures, Law 12546/2011 on tobacco-free environments and its regulation by Presidential Decree 8.262/2014 prohibits smoking indoors and regulates the exposure of cigarettes exclusively at points of sale, expanding the space occupied by sanitary warnings. Other measures included increasing cigarette taxation and setting the minimum price for tobacco, according to the best evidence for tobacco reduction [10, 34].

However, between 2015 and 2017, surveys, such as VIGITEL, point to the plateauing of smoking prevalence in the country, which may indicate regulatory and price policy failures [19] or the requirement of revision and improvement. Other studies have shown the increase in use of other tobacco products among adolescents, in particular the narghile, pointing to recent changes in the behavior of tobacco use in the country. Since 2015, Brazil has been undergoing a political and economic crisis, implementing measures of fiscal austerity, budget cuts, and less investment in public policies. Constitutional Amendment 95, approved by the Federal Government in 2016, has frozen the financial budgets of health, education, science, and technology, among other social policies for the next 20 years. In the coming years, these measures will impact the actions and services rendered by the Brazilian Unified Health System, contributing to the deterioration of the health of the population, besides resulting in an increase in poverty and extreme poverty [41]. Reflections on these policies can already be observed in the weakening of the regulatory role of the Brazilian government in the issue of protective measures, where, in the last two years, prices of tobacco products have remained unchanged, in addition to the lower inspection of tobacco products and the increase in illegal trade [42, 43]. New measures are needed to advance the regulatory process, such as the adoption of plain packaging, enforcement of smoke-free environments and places of sale, prevention of illegal smuggling, and investment in supporting small-scale farmers in cultures, among other strategies [43].

One limitation of this study is that the indicator used by GBD refers to the exposure of smoked tobacco and does not include other smokeless tobacco products and electronic cigarettes. However, these products in Brazil, according to the latest household national survey, correspond to less than 0.3% of use. Moreover, electronic cigarettes are banned in the country. Moreover, estimates are based on self-reported data, which can result in information bias. The RR values used to estimate PAFs may not be fully representative of all possible risk outcomes experienced by sex and age, and over time. Finally, data on the results of (TMREL) or minimal risk were available for younger populations, under 30 years of age; therefore, the attribution of overload was limited to age groups of 30 years of age or older [3].

## Conclusions

The present study points to the improvement of the indicators related to current smokers in Brazil throughout the analyzed period and, consequently, the mortality attributable to smoking, especially that caused by cardiovascular and cancer diseases. The reductions in prevalence confirmed the continued decline in smoking in the country. Brazil has adopted a set of regulatory measures and has implemented anti-tobacco policies, which explain these achievements, although some drawbacks have been seen lately.

There are still large regional differences in prevalence and mortality attributed to tobacco. A higher prevalence in states with a better SDI reveals a link to cultural aspects, whereas a higher prevalence among women was observed in tobacco-producing states. A higher prevalence in the states with the best SDI reveals an association with cultural and economic aspects, for example, high prevalence in the states of the Southern Region (Rio Grande do Sul, Parana and Santa Catarina), which are tobacco producers. However, in the states with the best SDI and a high tobacco prevalence, such as São Paulo and Rio Grande do Sul, although mortality rates attributable to smoking are still high, there have been more significant reductions due to better access to health services, revealing differences, and regional inequities.

Maintenance and advancement depends on the adoption of new regulatory policies, such as plain packaging, as well as support provided for small farmers to diversify their crops in order to achieve the UN SDG goals. Unfortunately, it is also important to emphasize that regulatory measures have not been prioritized in recent years.

## Supplementary information

**Additional file 1: Supplementary Table 1**. Number of deaths and age-standardized mortality rate by causes of death attributable to smoking for 1990 and 2017, and percent change of the mortality rate of the mortality rates in the period between 1990 and 2017 for Brazil. **Supplementary Table 2**. Values of the decomposition analysis of the change in the number of deaths attributable to smoking from 1990 to 2017, presented in Fig. 7a, as being due to risk exposure, total population growth, and population aging, for both sexes.

## Data Availability

All of the data we used in this article are publicly available online on the official website of Institute of Health Metrics and Evaluation (http://ghdx.healthdata.org/gbd-results-tool).

## Abbreviations

IBGE in Portuguese: Brazilian Institute of Geography and Statistics
CDC: Center for Disease Control
CRA: Comparative risk assessment
UFMG in Portuguese: Federal University of Minas Gerais
FCTC: Framework Convention on Tobacco Control
GATS: Global Adult Tobacco Survey
GBD: Global Burden of Disease
IHME: Institute for Health Metrics and Evaluation
PNAD in Portuguese: National Household Sample Survey
PNSN in Portuguese: National Survey of Nutrition and Health
NCD: Non-communicable diseases
PAF: Population-attributable fractions
RR: Relative risk
SDI: Socio-Demographic Index
ST-GPR: Spatiotemporal Gaussian process regression
SDG: Sustainable Development Goals
VIGITEL in Portuguese: Telephone Survey Surveillance System for Risk and Protective Factors for Chronic Diseases
TMREL: Theoretical minimum risk exposure level
TFR: Total fertility rate
UI: Uncertainty interval
UN: United Nations
WHO: World Health Organization
WHS: World Health Survey
